# Hypogalactosylation of immunoglobulin G in rheumatoid arthritis: relationship to HLA-DRB1 shared epitope, anticitrullinated protein antibodies, rheumatoid factor, and correlation with inflammatory activity

**DOI:** 10.1186/s13075-018-1540-0

**Published:** 2018-03-14

**Authors:** Christian Schwedler, Thomas Häupl, Ulrich Kalus, Véronique Blanchard, Gerd-Rüdiger Burmester, Denis Poddubnyy, Berthold Hoppe

**Affiliations:** 1 Department of Rheumatology and Clinical Immunology, Charité - Universitätsmedizin Berlin, Charitéplatz 1, 10117 Berlin, Germany; 2 Department of Biology, Chemistry and Pharmacy, Freie Universität Berlin, Takustraße 3, 14195 Berlin, Germany; 3 Institute of Transfusion Medicine, Charité - Universitätsmedizin Berlin, Charitéplatz 1, 10117 Berlin, Germany; 4 Institute of Laboratory Medicine, Charité - Universitätsmedizin Berlin, Augustenburger Platz 1, 13353 Berlin, Germany; 5 Department of Gastroenterology, Infectiology and Rheumatology, Charité - Universitätsmedizin Berlin, Hindenburgdamm 30, 12203 Berlin, Germany; 6 German Rheumatism Research Centre, Charitéplatz 1, 10117 Berlin, Germany; 7 Institute of Laboratory Medicine, Unfallkrankenhaus Berlin, Warener Straße 7, 12683 Berlin, Germany

**Keywords:** Rheumatoid arthritis (RA), Axial spondyloarthritis (axSpA), HLA-DRB1 shared epitope (HLA-DRB1 SE), Anticitrullinated protein antibodies (ACPA), Rheumatoid factor (RF), IgG hypogalactosylation, C-reactive protein (CRP), Disease Activity Score in 28 joints (DAS28)

## Abstract

**Background:**

Galactosylation of immunoglobulin G (IgG) is reduced in rheumatoid arthritis (RA) and assumed to correlate with inflammation and altered humoral immunity. IgG hypogalactosylation also increases with age. To investigate dependencies in more detail, we compared IgG hypogalactosylation between patients with RA, patients with axial spondyloarthritis (axSpA), and healthy control subjects (HC), and we studied it in RA on the background of HLA-DRB1 shared epitope (SE), anticitrullinated protein antibodies (ACPA), and/or rheumatoid factor (RF) status.

**Methods:**

Patients with RA (*n* = 178), patients with axSpA (*n* = 126), and HC (*n* = 119) were characterized clinically, and serum IgG galactosylation was determined by capillary electrophoresis. Markers of disease activity, genetic susceptibility, and serologic response included C-reactive protein (CRP), erythrocyte sedimentation rate (ESR), DAS28, SE, HLA-B27, ACPA, and RF. Expression of glycosylation enzymes, including beta 1–4 galactosyltransferase (B4GALT3) activity, were estimated from transcriptome data for B-cell development (GSE19599) and differentiation to plasma cells (GSE12366).

**Results:**

IgG hypogalactosylation was restricted to RA and associated with increasing CRP levels (*p* < 0.0001). In axSpA, IgG hypogalactosylation was comparable to HC and only marginally increased upon elevated CRP. Restriction to RA was maintained after correction for CRP and age. Treatment with sulfasalazine resulted in significantly reduced IgG hypogalactosylation (*p* = 0.003) even after adjusting for age, sex, and CRP (*p* = 0.009). SE-negative/ACPA-negative RA exhibited significantly less IgG hypogalactosylation than all other strata (vs SE-negative/ACPA-positive, *p* = 0.009; vs SE-positive/ACPA-negative, *p* = 0.04; vs SE-positive/ACPA-positive, *p* < 0.02); however, this indicated a trend only after Bonferroni correction for multiple testing. In SE-positive/ACPA-negative RA IgG hypogalactosylation was comparable to ACPA-positive subsets. The relationship between IgG hypogalactosylation and disease activity was significantly different between strata defined by SE (CRP, *p* = 0.0003, *p*_Bonferroni_ = 0.0036) and RF (CRP, *p* < 0.0001, *p*_Bonferroni_ < 0.0012), whereas ACPA strata revealed only a nonsignificant trend (*p* = 0.15). Gene expression data indicated that the key enzyme for galactosylation of immunoglobulins, B4GALT3, is expressed at lower levels in B cells than in plasma cells.

**Conclusions:**

Increased IgG hypogalactosylation in RA but not in axSpA points to humoral immune response as a precondition. Reduced B4GALT3 expression in B cells compared with plasma cells supports relatedness to early B-cell triggering. The differential influence of RA treatment on IgG hypogalactosylation renders it a potential diagnostic target for further studies.

**Electronic supplementary material:**

The online version of this article (10.1186/s13075-018-1540-0) contains supplementary material, which is available to authorized users.

## Background

**Fig. 1:**
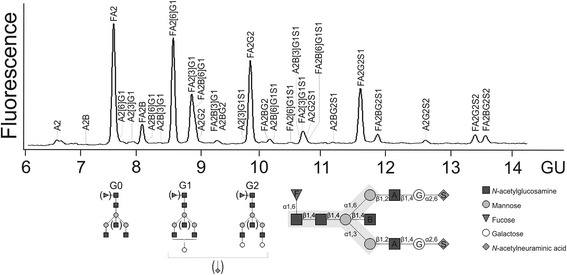
Immunoglobulin G (IgG) *N*-glycan structure and analysis. Composition of *N*-glycan structures attached to IgG. *N*-glycans share a common pentasaccharide core consisting of two *N*-acetylglucosamines (GlcNAc) and three mannose residues (*gray background*). Major structures are represented by agalactosylated IgG (G0), monogalactosylated IgG (G1), and digalactosylated IgG (G2) glycoforms. Above, a representative capillary electrophoresis laser-induced fluorescence detection electropherogram of fragment crystallizable region of IgG *N*-glycans is shown. *F* Core fucose, A*x* Number of antennary GlcNAc, *B* Bisecting GlcNAc linking beta 1–4 to beta 1–3 mannose, G*x* Number of beta 1–4 linked galactose (G), *[3]G1* and *[6]G1* galactose on antenna of alpha 1–3 or alpha 1–6 mannose, respectively, S*x* Number of *N*-acetylneuraminic acids linked to galactose, *GU* Glucose units

Rheumatoid arthritis (RA) is a common chronic inflammatory disease of unknown etiology [1]. Because many patients develop joint damage at an early stage after disease onset, it is of special clinical importance to identify and characterize the pathomechanisms in those patients who need early and aggressive therapy [2, 3]. B-cell involvement with production of rheumatoid factor (RF) or anticitrullinated protein antibodies (ACPAs) in about three-fourths of patients with RA significantly influences the clinical course, with more severe joint destruction and more cardiovascular complications [4, 5]. ACPA and RF are intimately connected to the pathogenesis of RA [6]. Based on this concept, the generation of ACPA or other antibodies against posttranslational modified antigens results in formation of immune complexes (ICs) that are involved in a first wave of inflammation, which is reflected by intraarticular complement activation. The generation of RF that is assumed to be slightly delayed in relation to ACPA results in the production of RF-ICs, which potently activate complement and correspond to a second wave of stronger inflammatory activity. Based on clinical data and on differences in the genetic background of ACPA-positive and ACPA-negative RA, the assumption is that the presence or absence of ACPA defines distinct RA subsets [1]. The genetic background for ACPA in response to different environmental triggers (e.g., smoking) primarily is given by the so-called human leukocyte antigen (HLA)-DRB1 shared epitope (SE) [7–11]. The fragment crystallizable regions (Fc) of ACPA and RF immunoglobulin G (IgG) antibodies carry complex oligosaccharides (Fig. 1) [12], which are structurally diverse and modulate the function and stability of IgG [13–16]. IgG *N*-glycan diversity has functional consequences (e.g., by modulating interaction with different receptors), thereby influencing antibody-dependent cell cytotoxicity and proinflammatory activity [17–24]. Some decades ago, RA was observed to be associated with reduced galactosylation of whole serum IgG [25, 26], a finding that since has been measured by the so-called serum G0/G1 ratio reflecting the relationship between agalactosylated immunoglobulin G (G0) and monogalactosylated immunoglobulin G (G1) [27]. This IgG hypogalactosylation precedes disease onset by several years and correlates with severity of RA [27–29]. Interestingly, IgG hypogalactosylation normalizes upon remission of RA during pregnancy or under effective treatment [30–33]. It has been described that IgG hypogalactosylation increases with age [17].

Several questions on IgG hypogalactosylation in RA are unaddressed. Comparing the time frame of changes in IgG galactosylation and C-reactive protein (CRP) in RA reveals a striking analogy in that IgG hypogalactosylation [27] and CRP elevations [34] precede RA onset by several years. Thus, the question arises whether IgG hypogalactosylation indicates specific processes involved in humoral autoimmunity or whether it simply reflects inflammatory activity. We addressed this question by analyzing IgG galactosylation in RA as well as in axial spondyloarthritis (axSpA). Spondyloarthritides represent chronic inflammatory diseases without relevant antibody reactivity and only moderate efficacy of B-cell depletion with rituximab [35, 36]. Because humoral autoimmunity in RA is influenced by HLA-DRB1 SE and is reflected by ACPA and/or RF synthesis, we tested whether these three factors are interrelated with IgG hypogalactosylation and whether the relationship between IgG hypogalactosylation and inflammatory activity differs in RA subsets defined by HLA-DRB1 SE, ACPA, or RF.

## Methods

### Patients and control subjects

Patients with RA were attending the Department of Rheumatology and Clinical Immunology (Berlin). All patients fulfilled American College of Rheumatology criteria for classification of RA [37]. Serum samples from 178 patients with RA were selected according to ACPA and/or SE status with other clinical characteristics blinded. Additionally, 126 patients with ankylosing spondylitis (AS; *n* = 64) or nonradiographic axial spondyloarthritis (nr-axSpA; *n* = 62) were included from the GErman SPondyloarthritis Inception Cohort, which was described previously [38]. Samples from blood donors at the Institute of Transfusion Medicine (Berlin) with a similar age and sex distribution as RA were used as healthy control subjects (HC). None of them presented with symptomatic autoimmune disorders or clinically detectable inflammatory or infectious disease. Baseline characteristics of the study population are summarized in Table 1 and Additional file 1: Table S1. All individuals were included in the study after they provided informed consent. The study was approved by the Charité institutional ethics committee.

**Table 1:** Baseline characteristics of patients and healthy controls

Characteristics	RA (*n* = 178)	axSpA (*n* = 126)	HC (*n* = 119)
Age, years, mean ± SD	55.0 ± 12.4	44.3 ± 8.4	55.2 ± 13.5
Female sex, *n* (%)	148 (78.3)	82 (65.1)	93 (78.2)
Disease duration, years, median (IQR)	5.4 (1.4–11.2)	4.5 (2.1–5.5)	NA
G0/G1 ratio, median (IQR)	1.25 (1.09–1.56)	0.89 (0.79–1.09)	0.95 (0.77–1.13)
CRP, mg/L, mean ± SD	19.4 ± 30.6^a^	5.8 ± 10.3	NA
DAS28, mean ± SD	5.1 ± 1.4^a^	NA	NA
ACPA-positive, *n* (%)	96 (53.9)	NA	NA
RF-positive, *n* (%)	136 (76.4)	NA	NA
HLA-DRB1 SE-positive, *n* (%)	96 (53.9)	NA	NA
HLA-B27-positive, *n* (%)	NA	88 (69.8)	NA
Previous/ongoing RA therapy, *n* (%)	127 (71%)	NA	NA

*Abbreviations: RA* Rheumatoid arthritis, *axSpA* Axial spondyloarthritis, *HC* Healthy control subjects, *G0/G1 ratio* Ratio of agalactosylated to monogalactosylated immunoglobulin G, *CRP* C-reactive protein, *DAS28* Disease Activity Score in 28 joints, *ACPA* Anticitrullinated protein antibody, *RF* Rheumatoid factor, *HLA* Human leukocyte antigen, *SE* Shared epitope, *NA* Not applicable

^a^ Data available in 173 patients

### Laboratory diagnostics and HLA-DRB1 genotyping

ACPA were quantified at the time of enrollment with a second-generation enzyme-linked immunosorbent assay (ELISA) using the CCPlus Immunoscan kit (Euro-Diagnostica, Malmö, Sweden) for detection of RA [39]. A cutoff value of 25 AU/ml was used. RF was quantified using an ELISA-based IgM-specific technique (cutoff value, 24 IU/ml) as previously described (DLD Diagnostika, Hamburg, Germany) [39]. CRP and erythrocyte sedimentation rate (ESR) were characterized using standard techniques [39]. SE was defined by HLA-DRB1 alleles with HLA-DRB1 chain residues 67Leu–69Glu–71Lys/Arg–74Ala–86Gly/Val and characterized using standard techniques (Dynal, Oslo, Norway; GenoVision, Vienna, Austria; PROTRANS, Ketsch, Germany) [39, 40].

### Glycan characterization

Analysis of IgG-Fc *N*-glycans was performed as described previously [41] with minor modifications. Briefly, IgG was isolated from human serum using protein A Sepharose. IgG-Fc glycopeptides were separated from IgG antigen-binding fragment by pepsin digestion and isolated by ultrafiltration. After denaturation, IgG-Fc *N*-glycans were released by peptide:N-glycosidase F (Roche Applied Science, Indianapolis, IN, USA) digestion, separated with reversed-phase Supelco SP20SS resin microcolumns (Sigma-Aldrich, St. Louis, MO, USA) and subsequently desalted with graphite microcolumns (Agilent Technologies, Santa Clara, CA, USA). *N*-glycans were derivatized by methylation of carboxylic acid groups of terminal sialic acids to neutralize their negative charges, followed by purification to remove excess reagents. Subsequently, dried eluates were labeled with 8-aminopyrene-1,3,6-trisulfonic acid (APTS) (Sigma-Aldrich) at 55 °C for 2 h in darkness. Capillary electrophoresis of labeled *N*-glycans was performed on a Beckman P/ACE MDQ system equipped with laser-induced fluorescence detection (Beckman Coulter, Brea, CA, USA). Separation was achieved with reversed polarity on polyvinyl alcohol-coated capillaries (50 μm inner diameter, 50-cm effective separation length from injection to detector; Beckman Coulter) using the background electrolyte (Beckman Coulter). APTS-labeled maltose was used as an internal standard to normalize detected migration time. Migration time was expressed in glucose units using an APTS-labeled dextran hydrolysate ladder. Electropherogram quality was evaluated by intensities and signal-to-noise ratio of peaks detected in all samples. Data analysis was performed with 32 Karat 8.0 software (Beckman Coulter). Structural assignment of IgG-Fc glycans was performed as reported previously. In this study 22 peaks were detected, which represent 29 glycan structures. Relative intensities were calculated after normalization as the ratio of areas between individual peaks to all peaks. In peaks with relevant comigration, the *N*-glycan structure of the dominant peak defined the galactosylation type. Glycosylation traits were quantified by summing the corresponding peak areas, where the abbreviations depict the number of antennary *N*-acetylglucosamine (GlcNAc) (A), bisecting GlcNAc (B), core fucose (F), galactose (G1, G2), and sialic acids (S1, S2) (Fig. 1): agalactosylation (G0) = A2 + A2B + FA2 + FA2B; monogalactosylation (G1) = A2[3]G1 + A2B[3]G1 + FA2[6]G1 + FA2[3]G1 + FA2B[6]G1 + FA2B[3]G1 + A2[3]G1S1 + FA2[6]G1S1 + A2B[3]G1S1 + FA2[3]G1S1; and G0/G1 ratio = (G0)/(G1). Because G0 and G1 represent over 70% of total IgG-Fc glycosylation, the IgG G0/G1 ratio was used to characterize aberrant IgG galactosylation as described previously for analyses on whole serum *N*-glycans as well as purified IgG [27]. Additionally, data on the main findings are presented for G0%, which represents the percentage of G0 among total IgG glycans.

### Analysis of beta 1–4 galactosyltransferase activity in B cells and plasma cells

Beta 1–4 galactosyltransferase (B4GALT3) activity was estimated from transcriptome data for B-cell development (Gene Expression Omnibus accession number [GEO:GSE19599]) and differentiation to plasma cells (Gene Expression Omnibus accession number [GEO:GSE12366]) using data from the Gene Expression Omnibus (GEO) online repository [42, 43]. The respective data of this repository were derived from two independently pooled samples of each cell population that have subsequently been hybridized onto Applied Biosystems GeneChip Human Genome U133 Plus 2.0 microarrays (Thermo Fisher Scientific, Waltham, MA, USA). Array data have been uploaded into the BioRetis database (www.bioretis.de), and normalized signal intensities as well as group analyses have been tested.

### Statistical analysis

Statistical analyses were performed using IBM SPSS Statistics 23.0 (IBM, Armonk, NY, USA) and Prism 6.0 software (GraphPad Software, La Jolla, CA, USA). The Kruskal-Wallis test followed by Dunn’s multiple comparisons test, the Wilcoxon rank-sum test, and the Mann-Whitney *U* test were used to analyze differences between groups. Associations between IgG G0/G1 ratio and disease-related parameters were evaluated by regression analysis. For secondary analyses (i.e., analyses of different IgG hypogalactosylation between RA subgroups defined by ACPA, RF, and SE as well as different relationships between IgG hypogalactosylation and activity markers in dependence of ACPA, RF, and SE), *p* values after Bonferroni correction for multiple testing assuming 12 secondary hypotheses are given.

## Results

### IgG G0/G1 ratio in RA, axSpA, and HC

**Fig. 2:**
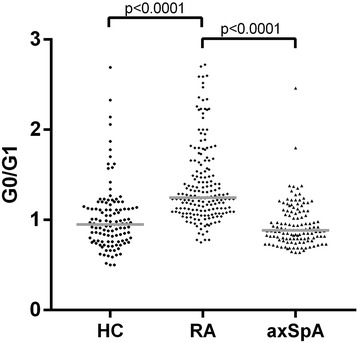
Immunoglobulin G (IgG) galactosylation in patients with rheumatoid arthritis (RA), patients with axial spondyloarthritis (axSpA), and healthy control subjects (HC). Comparison of agalactosylated immunoglobulin G/monogalactosylated immunoglobulin G (G0/G1) ratios in RA (*n* = 178), axSpA (*n* = 126), and HC (*n* = 119) is shown. *Horizontal lines* indicate median of distribution. *p* Values derived by Mann-Whitney *U* test are indicated

**Fig. 3:**
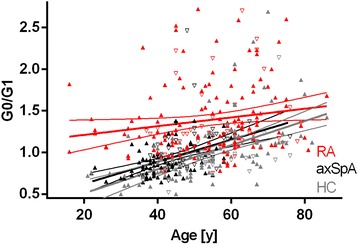
Immunoglobulin G (IgG) galactosylation in patients with rheumatoid arthritis (RA), patients with axial spondyloarthritis (axSpA), and healthy control subjects (HC) in dependency on age. Comparison of agalactosylated immunoglobulin G/monogalactosylated immunoglobulin G (G0/G1) ratios over age in RA (*n* = 178), axSpA (*n* = 126), and HC (*n* = 119) is shown. Results of regression analyses and 95% CIs are given. *p* < 0.001 for RA vs axSpA as well as RA vs HC. Data points for RA, axSpA, and HC are given in *red*, *black*, and *gray*, respectively; sex is indicated by *upright triangles* (female) and *inverted triangles* (male)

In patients with RA, IgG G0/G1 (median, 1.25; IQR, 1.09–1.56) was significantly higher than in HC (0.95; 0.77–1.13) (*p* < 0.0001) and axSpA (0.89; 0.79–1.09) (*p* < 0.0001) (Fig. 2). Whereas axSpA exhibited inflammatory activity as measured by elevated CRP level, in HC acute or recent inflammatory processes were excluded. The patients with axSpA did not differ from HC in terms of IgG G0/G1 ratios, supporting the hypothesis that inflammation is not a sufficient precondition for IgG hypogalactosylation. The RA cohort exhibited age and sex distributions similar to those of HC. However, the axSpA cohort differed considerably from RA and HC with respect to age and sex. Thus, analyses stratified by age as well as logistic regression analyses adjusting for age and sex as possible confounders were performed. We analyzed the differences in IgG G0/G1 ratio between RA and axSpA in age categories (≤ 25 years, 25–45 years, 45–65 years, > 65 years). In all age categories, RA exhibited higher IgG G0/G1 ratios than axSpA. These differences in IgG G0/G1 ratios reached statistical significance in patients aged 25–45 years (median [IQR], RA, 1.17 [1.00–1.29]; axSpA, 0.83 [0.73–0.95]; *p* < 0.0001) and 45–65 years (RA, 1.25 [1.09–1.59]; axSpA, 1.03 [0.85–1.21]; *p* < 0.0001). In the small group of patients aged ≤ 25 years (five patients with RA, two patients with axSpA), a nonsignificant trend was found (RA, 1.27 [1.23–1.40]; axSpA, individual IgG G0/G1 ratios 0.75 and 0.82; *p* = 0.053). In patients aged > 65 years (46 patients with RA, 2 patients with axSpA), the nonparametric comparison was nonsignificant (RA, 1.40 [1.16–1.79]; axSpA, individual IgG G0/G1 ratios 1.19 and 1.37; *p* = 0.66). When we compared RA, axSpA, and HC in a regression model, IgG hypogalactosylation was significantly more pronounced in RA than in axSpA and HC even after correction for age and sex as possible confounders (*p* < 0.001 for both comparisons). Plots for IgG G01/G1 ratio over age in all study cohorts are shown in Fig. 3.

An age- and sex-corrected comparison of G0% between RA and axSpA or HC confirmed significantly higher IgG hypogalactosylation in RA than in axSpA or HC (*p* < 0.001 for both comparisons; G0% median [IQR], RA, 41.5 [38.2–47.2]; HC, 35.4 [29.6–40.4]; axSpA, 32.5 [28.6–38.0]).

In the RA cohort, we tested whether disease duration would influence the IgG G0/G1 ratio. When patients were categorized in quartiles of disease duration (median [minimum–maximum], first quartile, 6 months [1 week–17 months]; second quartile, 2.6 years [1.5–5.2 years]; third quartile, 7.6 years [5.3–10.6 years]; fourth quartile, 16.6 years [10.9–60.8 years]), nonparametric testing revealed no trend for higher IgG G0/G1 ratio with increasing disease duration (*p* = 0.64). However, when the different quartiles of disease duration were considered separately, the first quartile exhibited a relatively high IgG G0/G1 ratio, followed by a significant decline in the second quartile and a re-increase of IgG G0/G1 ratio in the third and fourth quartiles (IgG G0/G1 ratio, median [IQR], first quartile, 1.36 [1.17–1.82]; second quartile, 1.19 [1.08–1.35]; third quartile, 1.2 [1.07–1.54]; fourth quartile, 1.31 [1–17–1.73]).

When patients with RA were differentiated by previously untreated and treated patients, no difference with respect to IgG hypogalactosylation could be found between groups (*p* = 0.74). When we looked at specific drugs, those patients treated with sulfasalazine (*n* = 47) exhibited significantly less IgG hypogalactosylation than patients treated without sulfasalazine (*n* = 131) (IgG G0/G1, median [IQR], treated with sulfasalazine, 1.16 [1.07–1.29]; treated without sulfasalazine, 1.31 [1.1–1.66], *p* = 0.001; G0%, treated with sulfasalazine, 40.1 [37.3–42.5]; treated without sulfasalazine, 42.9 [38.6–49.5], *p* = 0.003). This finding remained statistically significant after correction for age, sex, and CRP level (*p* = 0.009 for IgG G0/G1 ratio as well as G0%). In contrast, methotrexate, leflunomide, hydroxychloroquine, and tumor necrosis factor inhibitors did not exhibit altered IgG galactosylation.

### IgG G0/G1 ratio in RA and axSpA in relation to CRP level

**Fig. 4:**
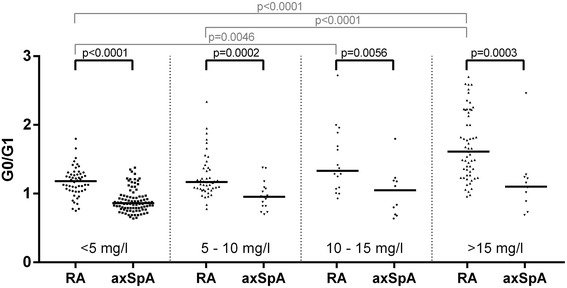
Immunoglobulin G galactosylation in patients with rheumatoid arthritis (RA) and patients with axial spondyloarthritis (axSpA) in different C-reactive protein (CRP)-level categories. Comparison of agalactosylated immunoglobulin G/monogalactosylated immunoglobulin G (G0/G1) ratios in RA (*n* = 173) and axSpA (*n* = 126) categorized by increasing CRP levels. *Horizontal lines* indicate the median of distribution. *p* Values derived by Mann-Whitney *U* test are indicated

**Fig. 5:**
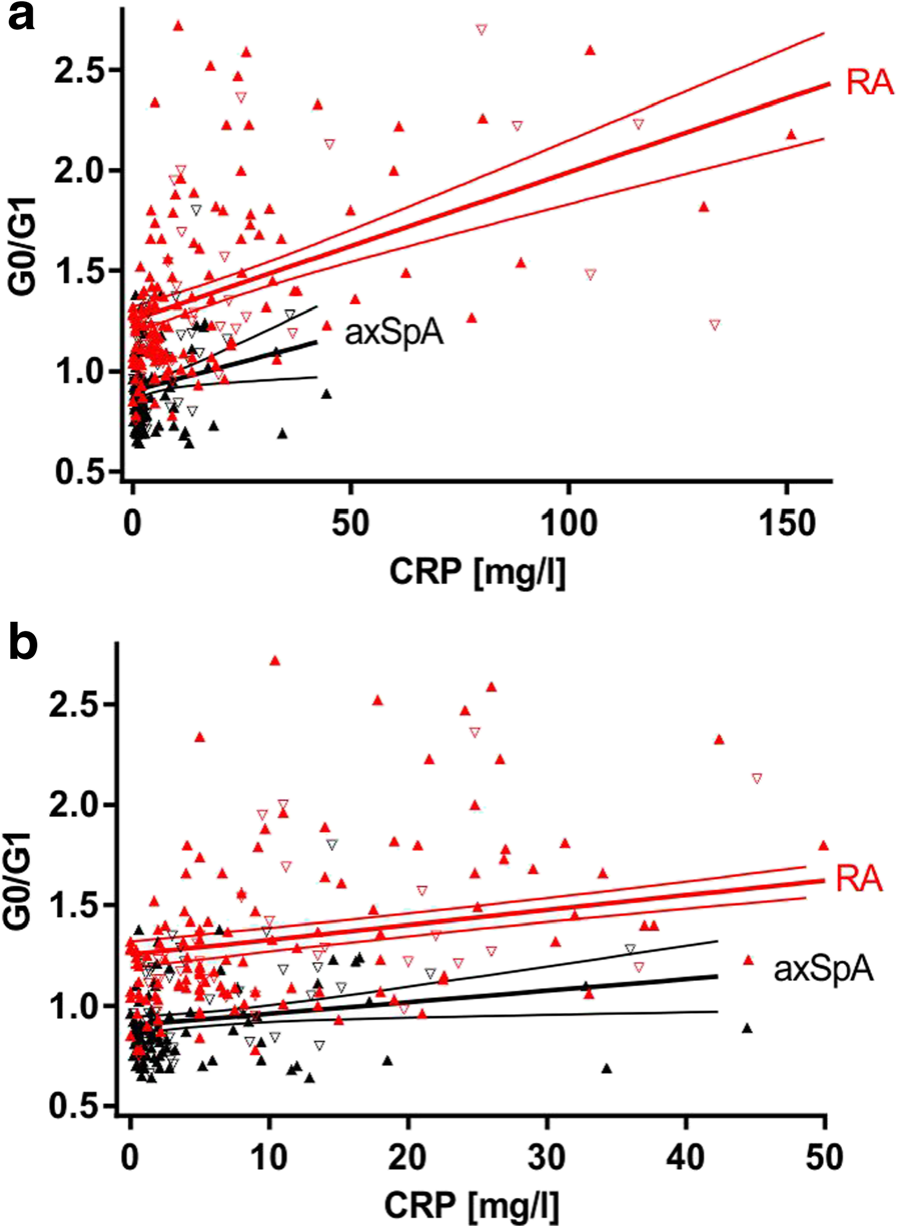
Immunoglobulin G galactosylation in patients with rheumatoid arthritis (RA) and patients with axial spondyloarthritis (axSpA) in dependency on C-reactive protein (CRP). Comparison of agalactosylated immunoglobulin G/monogalactosylated immunoglobulin G (G0/G1) ratios (**a**) over the complete CRP level or (**b**) over the CRP level < 50 mg/L in RA (*n* = 173) and axSpA (*n* = 126). Results of regression analyses and 95% CIs are given. *p* < 0.001 for RA vs axSpA. Data points for RA and axSpA are given in *red* and *black*, respectively; sex is indicated by *upright triangles* (female) and *inverted triangles* (male)

We compared RA and axSpA with respect to IgG G0/G1 ratio after stratifying for different CRP levels (< 5 mg/L, 5–10 mg/L, 10–15 mg/L, and > 15 mg/L) (Fig. 4). This analysis showed that across all CRP categories in patients with RA, significantly higher IgG G0/G1 ratios were present than in patients with axSpA (CRP < 5 mg/L, RA, 1.18 [1.04–1.29], vs axSpA, 0.86 [0.78–0.97], *p* < 0.0001; CRP 5–10 mg/L, RA, 1.17 [1.07–1.37], vs axSpA, 0.96 [0.82–1.09], *p* = 0.0002; CRP 10–15 mg/L, RA, 1.33 [1.08–1.79], vs axSpA, 1.05 [0.70–1.19], *p* = 0.0056; CRP > 15 mg/L, RA, 1.61 [1.27–2.20], vs axSpA, 1.10 [0.89–1.24], *p* = 0.0003). Furthermore, a significant increase in IgG G0/G1 ratio with increasing CRP levels could be observed in RA but not in axSpA (Fig. 4). The difference in IgG hypogalactosylation as measured by IgG G0/G1 ratio as well as G0% between RA and axSpA was significant after adjustment for possible confounders (CRP, age, and sex) (*p* < 0.001) (Fig. 5).

### IgG G0/G1 ratio in relation to ACPA, RF and HLA-DRB1 SE

**Fig. 6:**
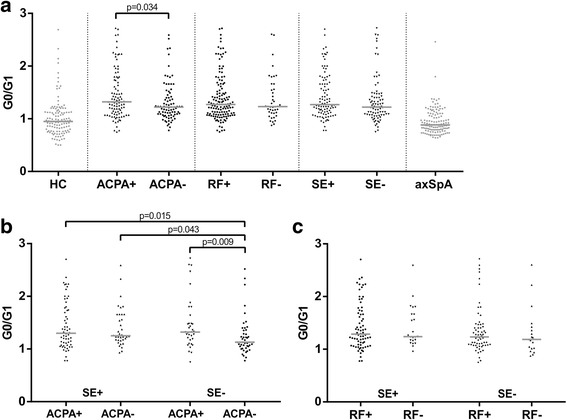
Immunoglobulin G (IgG) galactosylation in rheumatoid arthritis (RA), stratified by anticitrullinated protein antibody (ACPA), rheumatoid factor (RF) and human leukocyte antigen (HLA)-DRB1 shared epitope (SE) status. Comparison of agalactosylated immunoglobulin G/monogalactosylated immunoglobulin G (G0/G1) ratios in RA subsets defined by ACPA, RF, and HLA-DRB1 SE status. **a** IgG G0/G1 ratios in RA subsets defined by ACPA, RF, or SE status. **b** IgG G0/G1 ratios of RA subsets defined combinations of SE and ACPA status. **c** IgG G0/G1 ratios of RA subsets defined by combinations of SE and RF status. *Horizontal lines* indicate the median of distribution. *p* Values derived by Mann-Whitney *U* tests are indicated. In regard to multiple testing, a Bonferroni-corrected *p* value of *p* < 0.0042 should be applied. *axSpA* Axial spondyloarthritis, *HC* Healthy control subjects

The IgG G0/G1 ratio was tested in RA in dependency of the presence or absence of ACPA, RF, and SE. As shown in Fig. 6a, in ACPA-positive RA, the IgG G0/G1 ratio was significantly higher (1.32 [1.10–1.74]) than in ACPA-negative RA (1.22 [1.08–1.42]) (*p* = 0.034). This relatively small difference was significant in multivariate analyses adjusting for possible confounders (CRP, age, and sex) for IgG G0/G1 ratio (*p* < 0.02) and for G0% (*p* < 0.05; median [IQR], ACPA-positive, 42.9 [38.1–50.6]; ACPA-negative, 40.5 [38.3–44.9]). There was only a nonsignificant trend toward higher IgG G0/G1 ratio in HLA-DRB1 SE carriers (1.27 [1.12–1.68]) compared with noncarriers (1.22 [1.07–1.44]) (*p* = 0.09). IgG galactosylation in RF-positive (1.27 [1.08–1.57]) and RF-negative RA (1.23 [1.11–1.58]) was essentially the same (*p* = 0.81).

Subsequently, we studied IgG G0/G1 ratio in RA subsets defined by combinations of SE and ACPA status (Fig. 6b). Interestingly, in SE-positive/ACPA-negative RA, the IgG G0/G1 ratio (1.25 [1.17–1.66]) was largely comparable to both ACPA-positive subsets (ACPA-positive/SE-negative, 1.32 [1.17–1.64]; ACPA-positive/SE-positive, 1.30 [1.07–1.77]). In SE-negative/ACPA-negative RA, the IgG G0/G1 ratio (1.13 [1.05–1.35]) was significantly lower than in all other RA subsets. RA subsets defined by SE and RF status exhibited quite comparable IgG G0/G1 ratios (Fig. 6c). If considering a multiple testing strategy, Bonferroni correction would indicate only a trend.

### IgG G0/G1 ratio in axSpA subsets

**Fig. 7:**
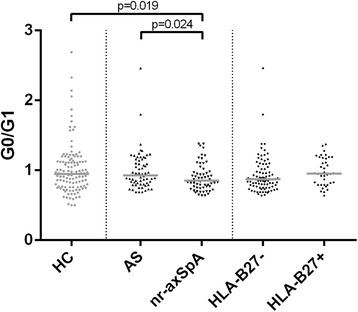
Immunoglobulin G (IgG) galactosylation in subgroups of axial spondyloarthritis (axSpA). Comparison of IgG agalactosylated immunoglobulin G/monogalactosylated immunoglobulin G (G0/G1) ratios in axSpA subsets defined ankylosing spondylitis (AS)/nonradiographic axial spondyloarthritis (nr-axSpA) or human leukocyte antigen (HLA)-B27 status. *Horizontal lines* indicate the median of distribution. *p* Values derived by Mann-Whitney *U* tests are indicated. *HC* Healthy control subject

The axSpA cohort was also evaluated considering two subgroups, AS and nr-axSpA (Fig. 7). In AS, we observed significantly higher IgG G0/G1 ratios (0.93 [0.81–1.16]) than in nr-axSpA (0.85 [0.75–0.97]) (*p* = 0.02). However, when adjusted for potentially confounding factors (sex and age), the difference in IgG G0/G1 ratio between AS and nr-axSpA was not statistically significant (*p* = 0.18). In addition, patients with nr-axSpA showed a significantly lower IgG G0/G1 ratio than HC (*p* < 0.02). Again, this difference did not survive adjustment for sex and age. There was a statistically nonsignificant trend toward lower IgG G0/G1 ratios in HLA-B27 carriers (0.87 [0.77–1.13]) than in noncarriers (0.96 [0.79–1.18]) (*p* = 0.07) (Fig. 7).

### Beta 1–4 galactosyltransferase 3 transcription in B cells and plasma cells

Representative transcriptomic data for comparison of the enzymatic activity involved in sugar modification of immunoglobulins were selected from the GEO database. Table 2 shows not only that transcripts for B4GALT3, the enzyme responsible for the beta 1–4 galactosylation of IgG, is expressed about threefold higher in plasma cells than in naive B cells but also that other transcripts of glycosyltransferases related to posttranslational modification of immunoglobulins are significantly increased in plasma cells compared with B cells.

**Table 2:**
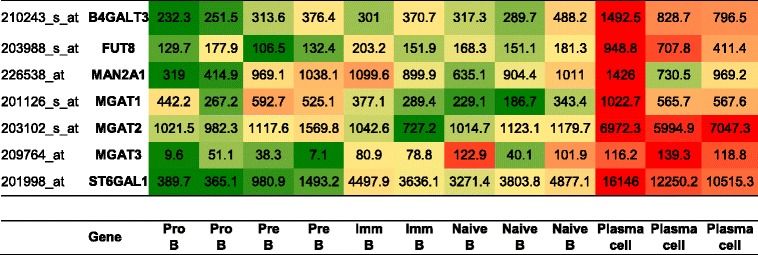
Analysis of glycosyltransferase transcription in B cells and plasma cells

Beta 1–4 galactosyltransferase (B4GALT3) transcription and transcription of other glycosyltransferases involved in immunoglobulin modification were estimated from transcriptomic data for B-cell development (Gene Expression Omnibus accession number [GSE19599]) and differentiation to plasma cells (Gene Expression Omnibus accession number [GSE12366]). Normalized signal intensities are given. Differentiation stages included are pro-B cells, pre-B cells, immature B cells, naive B cells, and plasma cells. Each column presents independent pools as described previously [42, 43]

### Relationship between IgG G0/G1 ratio and disease activity in RA

IgG G0/G1 ratio was correlated with Disease Activity Score in 28 joints (DAS28), CRP, and ESR (*p* < 0.0001 for all tests). Analyses regarding ACPA, RF, and SE status confirmed these results in all RA subsets (data not shown).

**Fig. 8:**
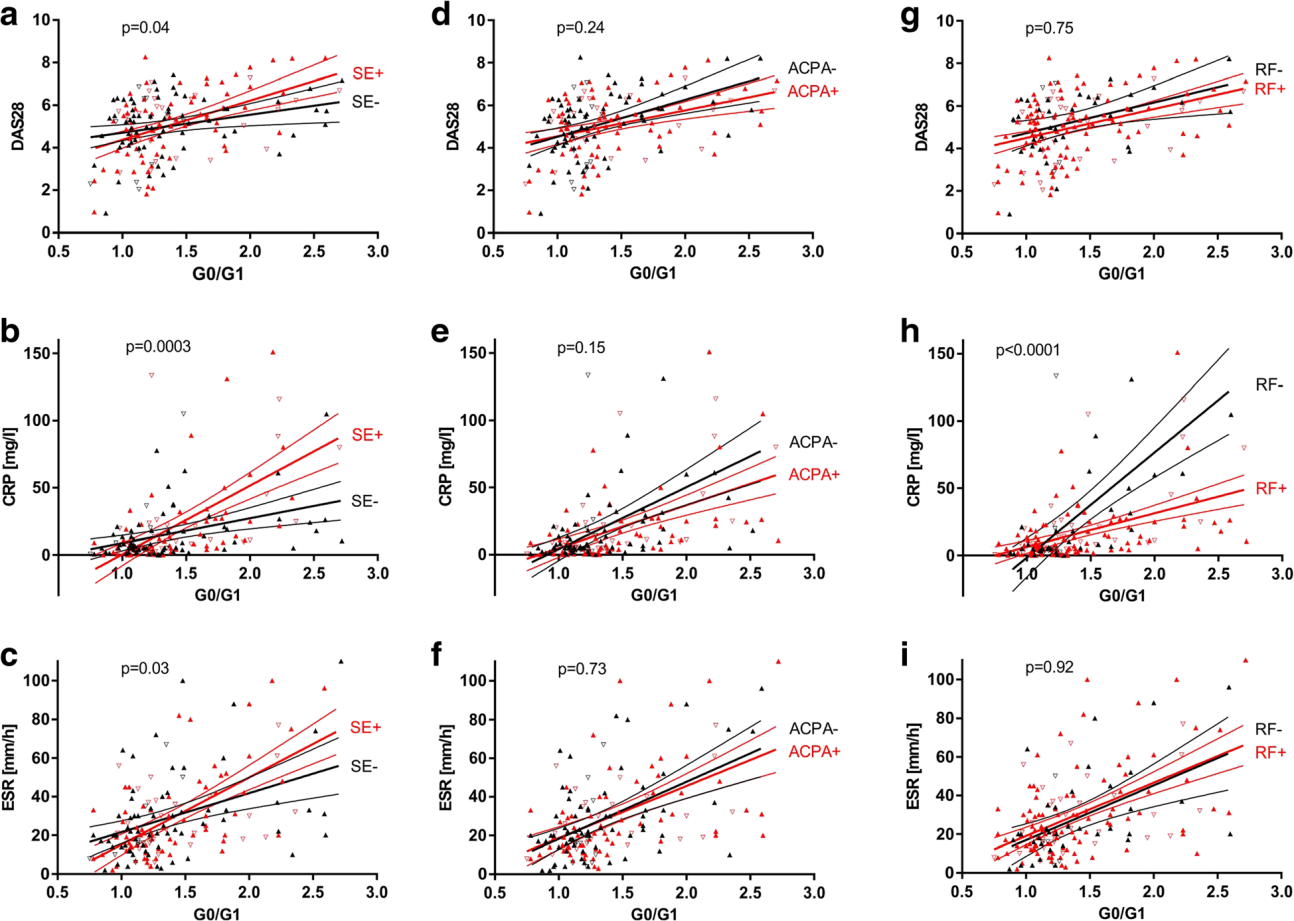
Correlation of immunoglobulin G (IgG) hypogalactosylation with disease activity in patients with rheumatoid arthritis (RA), stratified by human leukocyte antigen (HLA)-DRB1 shared epitope (SE), anticitrullinated protein antibody (ACPA), or rheumatoid factor (RF) status. Relationship between IgG agalactosylated immunoglobulin G/monogalactosylated immunoglobulin G (G0/G1) ratio and activity markers (Disease Activity Score in 28 joints [DAS28], C-reactive protein [CRP], and erythrocyte sedimentation rate [ESR]) in RA. Analyses for strata defined by HLA-DRB1 SE (**a–c**), ACPA (**d–f**), and RF (**g–i**) status. Results of regression analyses and 95% CIs are given, as well as *p* values for heterogeneity. Sex is indicated by *upright triangles* (female) and *inverted triangles* (male). In regard to multiple testing, a Bonferroni-corrected *p* value of *p* < 0.0042 should be applied

**Fig. 9:**
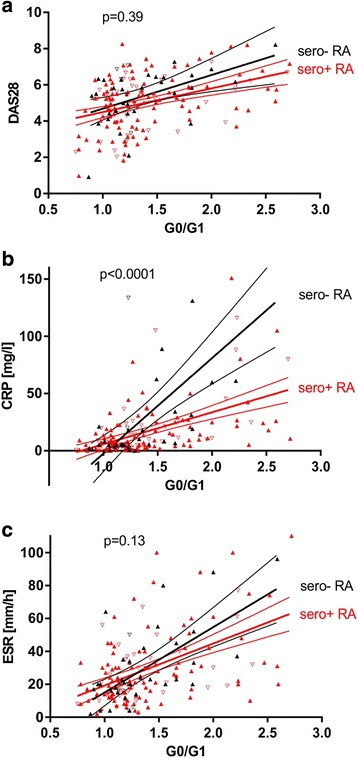
Correlation of immunoglobulin G (IgG) hypogalactosylation with disease activity in seropositive and seronegative rheumatoid arthritis (RA). Relationship between IgG agalactosylated immunoglobulin G/monogalactosylated immunoglobulin G (G0/G1) ratio and activity markers ((**a**) Disease Activity Score in 28 joints [DAS28], (**b**) C-reactive protein [CRP], and (**c**) erythrocyte sedimentation rate [ESR]) in RA, stratified by seropositive (sero+ anticitrullinated protein antibody [ACPA] and/or sero+ rheumatoid factor [RF]) and seronegative (sero− ACPA and sero− RF) status. Results of regression analyses and 95% CIs are given, as well as p values for heterogeneity. Sex is indicated by *upright triangles* (female) and *inverted triangles* (male). In regard to multiple testing, a Bonferroni-corrected *p* value of *p* < 0.0042 should be applied

When we compared the correlation between IgG G0/G1 ratio and RA disease activity (DAS28, CRP, and ESR) stratified by SE, ACPA, or RF, we identified a significant heterogeneity with respect to the presence or absence of SE and RF (Fig. 8). Stratified by SE, the correlations between IgG G0/G1 ratio and DAS28 (*p* = 0.04, *p*_Bonferroni_ = 0.48), CRP (*p* = 0.0003, *p*_Bonferroni_ = 0.0036), or ESR (*p* = 0.03, *p*_Bonferroni_ = 0.36) were significantly different in SE carriers and noncarriers. In general, HLA-DRB1 SE carriers exhibited a stronger correlation of IgG G0/G1 ratio with disease activity than noncarriers (Fig. 8a–c). Surprisingly, when we assessed these relationships between ACPA-positive and ACPA-negative RA, only a nonsignificant trend for heterogeneity could be detected (Fig. 8d–f). When we considered RF status, the correlation between IgG G0/G1 ratio and CRP was significantly stronger in RF-negative than in RF-positive RA (*p* < 0.0001, *p*_Bonferroni_ < 0.0012) (Fig. 8g–i). When we classified patients as seronegative (RF-negative and ACPA-negative) or seropositive (RF-positive and/or ACPA-positive), the relationship between IgG G0/G1 ratio and CRP was significantly stronger in seronegative than in seropositive RA (*p* < 0.0001, *p*_Bonferroni_ < 0.0012) (Fig. 9).

## Discussion

In this study, IgG hypogalactosylation was found to be related to RA, whereas axSpA presented with levels comparable to HC (Fig. 2). Inflammatory activity as measured by CRP was related to increased IgG hypogalactosylation in RA but had only marginal effects in axSpA (Figs. 4 and 5). IgG hypogalactosylation was higher in RA than in axSpA and HC when age was considered as a confounding factor, with a tendency toward convergence after the sixth decade of life (Fig. 3). Overall, there was no trend for a continuous increase of IgG hypogalactosylation with increasing disease duration.

Hypogalactosylation of IgG in RA was first observed decades ago and is also found in other chronic inflammatory conditions [44]. Whether this phenomenon is related solely to aberrant *N*-glycan formation during IgG synthesis [45] or to the use of alternative B-cell/plasma cell subsets [46], or whether altered *N*-glycan degradation makes some contribution, is unknown [47–49]. The increase of IgG agalactosylated glycoforms in RA has been discussed as being directly associated with reduced galactosyltransferase (GTase) activity in lymphocytes [47]. Previous studies have shown a possible mechanism for posttranslational regulation of GTase activity in RA. Thus, in RA, either qualitative and quantitative changes of GTase expression could result in altered enzymatic activity or the activity of specific B-cell/plasma cell subsets with preference for agalactosylated IgG is increased in RA [46].

Our findings suggest that triggering of the humoral response is necessary for changes in IgG galactosylation, or at least that inflammation itself is not a sufficient precondition in this respect. This is further supported by transcriptomic data (Table 2) that show that B cells, before differentiating to plasma cells, express lower levels of enzymes involved in glycosylation of immunoglobulins, in particular B4GALT3, the key enzyme of IgG galactosylation. This supports the assumption of increased IgG hypogalactosylation in newly triggered humoral response. The phenomenon of IgG hypogalactosylation in our study could be overlaid by age, sex, and inflammation related effects in all three groups, RA, axSpA and HC.

The finding that patients treated with sulfasalazine showed significantly decreased IgG hypogalactosylation compared with those without sulfasalazine treatment seemed surprising because the specific mode of therapeutic action of this drug is mainly unknown. However, this result is of striking compatibility with an earlier report describing reduced lymphocytic GTase activity in patients with RA and a re-increase in those patients treated with sulfasalazine [50]. On the basis of the information in this earlier report to IgG galactosylation, sulfasalazine treatment should reduce the amount of unoccupied potential galactosylation sites, thereby reducing IgG G0/G1 ratio and G0%, which is completely in agreement with our findings.

When looking for the influence of SE, ACPA, and RF on IgG hypogalactosylation (Fig. 6), SE-negative/ACPA-negative RA exhibited significantly less IgG hypogalactosylation than all other RA subsets, making this specific RA subset comparable to axSpA or HC from a glycophenotypic viewpoint. However, considering the Bonferroni correction for multiple testing qualifies these findings as trends, which should be addressed in further studies. On the other hand, IgG hypogalactosylation in SE-positive/ACPA-negative RA was at a level comparable to that in both ACPA-positive strata.

Correlation between IgG hypogalactosylation and inflammatory activity in RA has been described previously [27, 29, 51, 52]. Whether this relationship is due to an increased proinflammatory potential of hypogalactosylated IgG, or whether hypogalactosylation of IgG simply reflects an altered cellular activity or a shift in respective B-cell compartments (e.g., plasma cell, memory cell), is currently unknown. In our present study, the relationship between IgG hypogalactosylation and disease activity markers (Figs. 8 and 9) was dependent on SE, RF, and, with a statistically nonsignificant trend, on ACPA. The findings on the interrelationship of inflammatory activity and IgG hypogalactosylation could be interpreted as being that in seropositive RA, IgG hypogalactosylation is associated with lower CRP levels, or, in a more functional description, less inflammatory activity seems to be needed to induce aberrant IgG galactosylation. It could be considered that RF and ACPA may attenuate proinflammatory activity by capturing and eliminating the involved antigens as ICs, thereby interfering with a possible direct triggering of antigen-presenting cells and interleukin 6 as well as subsequent CRP production. Finally, association of IgG hypogalactosylation in HLA-DRB1 SE-positive patients with higher CRP levels suggests that antigen presentation in the context of SE is associated with a stronger proinflammatory immune activation prior to the involvement and differentiation of B cells, which seem to produce less IgG-galactosylating enzymes than plasma cells and thus would generate more hypogalactosylated IgG. These steps may depend on the extent of monocyte involvement, which contributes to the chronic inflammatory response by increased production, preterm release from bone marrow, reduced circulation time, and activation in the joint as reported recently [53]. Furthermore, HLA-DRB4, which is preferentially connected to the HLA-DRB1 SE, may exhibit differential effects on immune activation [54]. However, it should be mentioned that these interpretations are hypothetical and that the findings reported in Figs. 8 and 9 are based on analyses that could be affected by outliers and thus should be confirmed in independent studies.

The finding that in ACPA-positive RA the IgG G0/G1 ratio was significantly higher than in ACPA-negative RA (Fig. 6a), albeit losing statistical significance after correction for multiple testing, has been described previously when comparing purified anticitrullinated antibodies with repertoire IgG [27] and could be due to reduced galactosylation of ACPA. This finding would confirm the assumption that aberrant IgG galactosylation is related to altered humoral immunity [29]. Interestingly, RF status did not influence IgG G0/G1 ratio (Fig. 6a). A possible explanation could be that in RF-positive individuals, the amount of activated cell types with a preference for agalactosylated IgG is lower than in ACPA-positive individuals.

Some limitations of our study have to be mentioned. Owing to multiple testing, some findings are at risk for false-positive reporting. Our primary hypothesis was the assumption that inflammation itself is not a sufficient precondition for IgG hypogalactosylation. We think that this hypothesis is supported by our data at a sufficiently low significance level without as well as with correction for potentially confounding factors. Other findings that are based on relatively small patient subgroups should be confirmed in independent validation cohorts in the future.

## Conclusions

Our data support the assumption that inflammation itself is not a sufficient precondition for aberrant IgG galactosylation and that IgG hypogalactosylation in RA is reduced by specific treatments. Whether pretreatment IgG hypogalactosylation status would help in selection of specific treatment strategies or whether assessment of IgG hypogalactosylation during therapy could help with monitoring treatment responses would be an interesting topic for further studies.

## Additional files

Additional file 1: **Table S1**. Baseline characteristics of patient subgroups. (DOCX 14 kb)

## Abbreviations

ACPA: Anticitrullinated protein antibody
AS: Ankylosing spondylitis
axSpA: Axial spondyloarthritis
B4GALT3: Beta 1–4 galactosyltransferase 3
CRP: C-reactive protein
DAS28: Disease Activity Score in 28 joints
ELISA: Enzyme-linked immunosorbent assay
ESR: Erythrocyte sedimentation rate
Fc: Fragment crystallizable region
G0: Agalactosylated immunoglobulin G
G1: Monogalactosylated immunoglobulin G
G2: Digalactosylated immunoglobulin G
GEO: Gene Expression Omnibus repository
GlcNAc: *N*-acetylglucosamine
GTase: Galactosyltransferase
HC: Healthy control subject
HLA: Human leukocyte antigen
IC: Immune complex
IgG: Immunoglobulin G
nr-axSpA: Nonradiographic axial spondyloarthritis
RA: Rheumatoid arthritis
RF: Rheumatoid factor
SE: Shared epitope
