# Supplementary material for: Hypogalactosylation of immunoglobulin G in rheumatoid arthritis: relationship to HLA-DRB1 shared epitope, anticitrullinated protein antibodies, rheumatoid factor, and correlation with inflammatory activity
Source: Arthritis Res Ther. 2018 Mar 14;20:44. doi: 10.1186/s13075-018-1540-0 (PMC5853146; doi:10.1186/s13075-018-1540-0)
Supplement: Supplementary file 1 — Table S1. Baseline characteristics of patient subgroups. (DOCX 14 kb) [file 13075_2018_1540_MOESM1_ESM.docx]

**Supplemental table 1.** Baseline characteristics of patient subgroups

| Characteristics | ACPA pos.  (n = 95)  (*n= 94) | ACPA neg.  (n = 83)  (*n = 79) | RF pos.  (n = 136)  (*n = 132) | RF neg.  (n = 42)  (*n = 41) | SE pos.  (n = 96)  (*n = 96) | SE neg.  (n = 82)  (*n = 77) | AS  (n = 64) | nr-axSpA  (n = 62) |
| --- | --- | --- | --- | --- | --- | --- | --- | --- |
| Age [years], mean ± SD | 55.3 ± 12.3 | 54.6 ± 14.9 | 55.7 ± 13.2 | 52.6 ± 14.5 | 53.8 ± 13.2 | 56.3 ± 13.9 | 44.9 ± 9.5 | 43.6 ± 7.1 |
| Female sex, n (%) | 69 (72.6) | 70 (84.3) | 103 (75.7) | 36 (85.7) | 69 (71.9) | 70 (85.4) | 29 (45.3) | 53 (85.5) |
| Disease duration [years], median (IQR) | 6.2 (2.2-12.3) | 5.2 (0.9-10.2) | 5.9 (1.9-11.2) | 2.9 (0.5-10.1) | 4.6 (1.1-10.5) | 5.9 (1.6-11.3) | 5.3 (3.3-7.5) | 3.3 (1.3-5.4) |
| G0/G1 ratio, median (IQR) | 1.32 (1.10-1.74) | 1.22 (1.08-1.40) | 1.27 (1.08-1.54) | 1.23 (1.08-1.40) | 1.27 (1.12-1.66) | 1.22 (1.09-1.36) | 0.93 (0.80-1.14) | 0.85 (0.75-0.96) |
| CRP [mg/L], mean ± SD | 19.4 ± 28.2* | 19.0 ± 33.5* | 16.2 ± 23.4* | 29.2 ± 46.1* | 22.3 ± 36.1* | 15.3 ± 21.3* | 7.8 ± 13.1 | 3.7 ± 5.8 |
| DAS28, mean ± SD | 5.1 ± 1.4* | 5.1 ± 1.5* | 5.0 ± 1.5* | 5.3 ± 1.5* | 5.2 ± 1.6* | 5.0 ± 1.4* | NA | NA |
| ACPA positive, n (%) | 95 | 0 | 92 (67.6) | 3 (7.1) | 60 (62.5) | 35 (42.7) | NA | NA |
| RF positive, n (%) | 92 (96.8) | 44 (53.0) | 136 | 0 | 73 (76.0) | 63 (76.8) | NA | NA |
| HLA-DRB1 SE positive, n (%) | 60 (63.2) | 36 (43.4) | 58 (42.6) | 23 (54.8) | 96 | 0 | NA | NA |
| HLA-B27 positive, n (%) | NA | NA | NA | NA | NA | NA | 45 (70.3) | 43 (69.4) |

G0/G1 ratio: ratio of agalactosylated (G0) to monogalactosylated IgG (G1), CRP: C-reactive protein, DAS28: Disease Activity Score in 28 joints, ACPA: anti-citrullinated protein antibody, RF: rheumatoid factor, HLA-DRB1 SE: HLA-DRB1 shared epitope, HLA-B27: human leucocyte antigen B27.
